# Western Medical Classes

**Published:** 1829-02

**Authors:** 


### WESTERN MEDICAL CLASSES.

*Medical College of Ohio.* The pupils of this institution during the present session amount to 113.

*Transylvania University.* The number of pupils in the medical department of this institution, at the present time, is 203.

